# Nuclear factor-κB activation by transforming growth factor-β1 drives tumour microenvironment-mediated drug resistance in neuroblastoma

**DOI:** 10.1038/s41416-024-02686-8

**Published:** 2024-05-28

**Authors:** Kévin Louault, Laurence Blavier, Men-Hua Lee, Rebekah J. Kennedy, G. Esteban Fernandez, Bruce R. Pawel, Shahab Asgharzadeh, Yves A. DeClerck

**Affiliations:** 1 Cancer and Blood Diseases Institute, Department of Pediatrics, Children’s Hospital Los Angeles and the University of Southern California, Los Angeles, CA 90027 USA; 2 The Saban Research Institute, Children’s Hospital Los Angeles, Los Angeles, CA 90027 USA; 3 Department of Pathology, and Laboratory Medicine, Children’s Hospital Los Angeles and Keck School of Medicine, University of Southern California, Los Angeles, CA 90033 USA; 4 Department of Biochemistry and Molecular Medicine, University of Southern California, Los Angeles, CA 90033 USA

**Keywords:** Cancer microenvironment, Paediatric cancer

## Abstract

**Background:**

Intrinsic and extrinsic factors in the tumour microenvironment (TME) contribute to therapeutic resistance. Here we demonstrate that transforming growth factor (TGF)-β1 produced in the TME increased drug resistance of neuroblastoma (NB) cells.

**Methods:**

Human NB cell lines were tested in vitro for their sensitivity to Doxorubicin (DOX) and Etoposide (ETOP) in the presence of tumour-associated macrophages (TAM) and mesenchymal stromal cells/cancer-associated fibroblasts (MSC/CAF). These experiments were validated in xenotransplanted and primary tumour samples.

**Results:**

Drug resistance was associated with an increased expression of efflux transporter and anti-apoptotic proteins. Upregulation was dependent on activation of nuclear factor (NF)-κB by TGF-β-activated kinase (TAK1) and SMAD2. Resistance was reversed upon pharmacologic and genetic inhibitions of NF-κB, and TAK1/SMAD2. Interleukin-6, leukaemia inhibitory factor and oncostatin M were upregulated by this TGF-β/TAK1/NF-κB/SMAD2 signalling pathway contributing to drug resistance via an autocrine loop activating STAT3. An analysis of xenotransplanted NB tumours revealed an increased presence of phospho (p)-NF-κB in tumours co-injected with MSC/CAF and TAM, and these tumours failed to respond to Etoposide but responded if treated with a TGF-βR1/ALK5 inhibitor. Nuclear p-NF-κB was increased in patient-derived tumours rich in TME cells.

**Conclusions:**

The data provides a novel insight into a targetable mechanism of environment-mediated drug resistance.

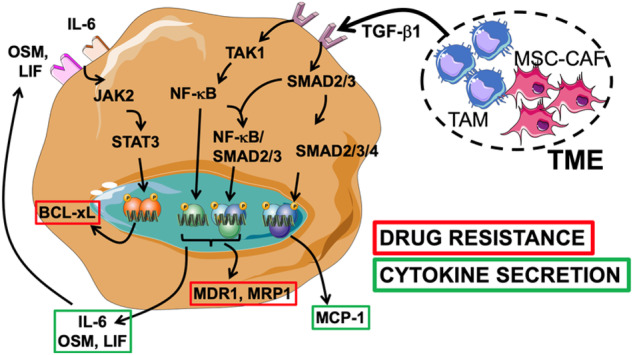

## Introduction

Drug resistance is a major cause of failure to cure patients affected with cancer. It is commonly the result of inherent characteristics of cancer cells linked to genomic and chromosomal instabilities and epigenetic alterations associated with selective pressure and tumour heterogeneity [1]. However, it can also be the result of external factors such as abnormal blood flow and influences from the tumour microenvironment (TME), where it is described as environment-mediated drug resistance (EMDR) [2].

Neuroblastoma (NB) is the most common extracranial solid tumour in children and a cancer with a high risk of recurrence [3–5]. Approximately fifty percent of the children diagnosed with NB are classified as having a high-risk cancer based on the stage of disease and biological features, including amplification of the MYCN oncogene (MYCN-A), loss or gain of genetic material, age at diagnosis, and presence of unfavourable histological features. Despite an intensive treatment with a combination of surgery, myeloablative chemotherapy, radiation therapy and immunotherapy, more than 50% of these children succumb to refractory or recurrent disease [6, 7]. The underlying mechanisms of chemotherapy resistance in NB are complex. One of the most common mechanisms occurs via the overexpression of ATP-binding cassette (ABC) efflux transporter proteins such as *ABCB1,* P-glycoprotein/multidrug resistant (P-gp/MDR1) and *ABCC1* multidrug resistance-associated protein (MRP)-1 that transport drugs like ETOP, DOX, Vincristine and Irinotecan outside the cell [8–12]. Other mechanisms involve the overexpression of anti-apoptotic proteins like survivin, BCL2, MCL-1 and BCL-xL [13].

We have previously reported that mesenchymal stromal cells (MSC) and cancer-associated fibroblasts (CAF) induce EMDR in NB through IL-6 and STAT3 [14–17]. CAF and their precursor MSC along with tumour-associated macrophages (TAM) are among the most abundant host non-malignant cells in the TME of NB [18]. They are often present together and their presence is typically a sign of unfavourable outcome [19, 20]. Upon interaction with NB cells, they release several pro-tumourigenic cytokines and chemokines, such as transforming growth factor (TGF)**-**β1, IL-6, IL-8 and macrophage chemoattractant protein-1 (MCP-1 a.k.a CCL-2) driving a Th-2 inflammatory reaction leading toward immune escape [18].

Here, we investigated how MSC and TAM in the TME induce EMDR in NB through TGF-β1.

## Materials and methods

### Cell lines and human monocytes

Human NB cell lines CHLA-255 (MYCN-NA and c-Myc expressing), SK-N-AS (MYCN-NA), SK-N-SH (MYCN-NA), CHLA-136 (MYCN-A), SK-N-BE(2) (MYCN-A) were established at Children’s Hospital Los Angeles and cultured as previously described [18]. Cell line authentication was done by genotype analysis using AmpFISTR® Identifier® PCR Amplification Kit and Gene Mapper ID v3.2 (Applied Biosystems). Some cell lines have been transfected with a Luciferase expression vector. Human MSC from the bone marrow of healthy donors were obtained from American Tissue Type Collection (ATCC) and cultured as previously described [18]. Human monocytes (MN) were isolated from the peripheral blood of healthy blood donors using a protocol previously reported [21]. A freshly purified MN preparation from different donors was used for each experiment. MN were routinely tested for viability by trypan blue exclusion and for their ability to differentiate into M1 macrophages in the presence of granulocyte-macrophage colony-stimulating factor (GM-CSF). TAM and CAF were obtained by culturing human MN or human MSC in the presence of NB cells for 72 h [18]. Cells were periodically assessed for the absence of mycoplasma using MycoAlert™ Plus Mycoplasma Detection Kit (Lonza).

### In vitro cultures

Co-culture experiments were performed in transwell (0.4 µm pore membranes, Corning). Dual co-cultures were performed with a 1:1 ratio for NB:MN and a 4:1 ratio for NB:MSC. Triple co-cultures had a 4:4:1 ratio for NB:MN:MSC. NB cells and MSC were seeded separately the first day and fresh MN were seeded after 24 h in the presence of Iscove’s Modified Dulbecco’s Medium (IMDM, Life Technologies) and 2% acid-treated foetal bovine serum (Ac-FBS) (v/v). Acid treatment of FBS was done to eliminate α2-macroglobulin (an inhibitor of TGF-β1 activation), by adding 10% 1 N HCl (v/v) for 10 min before the addition of 10% (v/v) 1.2 N NaOH-0.5 M HEPES to reach a pH of 7.4 (R&D Systems). Cultures were maintained for different periods of time depending on the experiments (10 min to 3 days). Cultures were treated with different concentrations of drugs or human recombinant (r)TGF-β1 (Supplemental Table 1) as indicated in the legend to the figures.

### Drug sensitivity assays

Neuroblastoma cells were seeded (1.5  ×  10^5^ cells/3.8 cm^2^ well) in the presence or absence of MN and/or MSC; or 1500 pg/ml of recombinant TGF-β1 (rTGF-β1) during 24 h, then treated with different drugs at concentrations ranging from 0 to 20 μM during 72 h (Supplementary Table 1). Cell survival was determined by trypan blue exclusion with three biological replicates for each cell line, with two wells and three counts per well, per condition.

### Enzyme-linked immunosorbent assay (ELISA) assays

Levels of TGF-β1, IL-6, MCP-1 (CCL-2), leukaemia inhibitory factor (LIF) and oncostatin M (OSM) in the conditioned-media (CM) were determined by ELISA using the Duo Set ELISA kit (R&D Systems) according to the manufacturer’s protocol. Each experiment was done on three to five biological replicates (two or three biological replicates by cell lines), with technical duplicate samples (two duplicates for two different dilutions). Samples were diluted if the initial values obtained were outside the linear standard curve.

### Western blot (WB) analysis

Cells were suspended in Radioimmunoprecipitation assay (RIPA) lysis buffer supplemented with a protease and phosphatase inhibitor cocktail (Thermo Fisher) according to the manufacturer’s protocol. Cell lysates were clarified by centrifugation (14,000 × *g*, 15 min, 4 °C). Total protein determination was performed using a Bicinchoninic acid (BCA) assay (Bio-Rad). Proteins (15–30 µg) were separated by sodium dodecyl sulfate polyacrylamide gel electrophoresis (SDS-PAGE) under reducing conditions using 4–15% (w/v) gradient Tris-Glycine gels (TGX^TM^, Bio-Rad) and transferred onto nitrocellulose membranes (Bio-Rad). Membranes were blocked for 2 h in PBS containing 10% (w/v) bovine serum albumin (Sigma) and 0.1% (v/v) Tween 20 and incubated with primary antibodies (Supplementary Table 2) overnight at 4 °C. After washing in phosphate buffered saline (PBS)-0.1% (v/v) Tween 20, membranes were incubated with IRDye infrared conjugated antibodies for 1 h at room temperature and detected using the Odyssey Imager system equipped with Image Studio V3.1 software (LI-COR Biosciences). Quantification of the WB was obtained using the ImageJ software (National Institutes of Health, NIH). Phosphoproteins were normalised to total proteins and other proteins were normalised to glyceraldehyde 3-phosphate dehydrogenase (GAPDH). Data were graphed in GraphPad Prism. Each experiment was done on three to five biological replicates from different cell lines.

### Co-immunoprecipitation (co-IP)

Neuroblastoma cells were cultured with MN and MSC (triple co-culture) or in the presence of 1500 pg/ml of rTGF-β1, in the presence or in the absence of Galunisertib during 72 h. NB cells were suspended in RIPA lysis buffer supplemented with a protease and phosphatase inhibitor cocktail and cell lysates were clarified by centrifugation (14,000 × *g*, 15 min, 4 °C). Co-IP of NB cells lysates was performed according to the manufacturer’s protocol (Protein G immunoprecipitation, catalogue number #IP50, Sigma). In brief, lysates were incubated with an anti-SMAD2/3 immunoprecipitating antibody (Supplemental Table 2) for 2 h at 4 °C under rotation. After addition of Protein G beads, the protein complexes were pelleted by centrifugation, washed with buffer (1 × IP Buffer) and incubated overnight at 4 °C under constant rotation. The protein complexes were pelleted by centrifugation (12,000 × *g*, 30 s), washed with IP buffer containing 0.5 M NaCl/0.1% SDS twice, and with IP buffer a fourth time, and analysed by SDS-PAGE and WB. Proteins were identified by immunoblotting with anti-NF-κB antibodies (Supplemental Table 2). The control used for co-IP was 10% (v/v) of the NB lysates to check the presence of the protein. Each experiment was done on three biological replicates on two different cell lines.

### Nuclear extraction

Neuroblastoma cells were collected with trypsin buffer and pelleted by centrifugation. Nuclear extraction was performed according to the manufacturer’s protocol (Nuclear extraction kit, catalogue number #2900, Millipore). In brief, NB cell pellets were resuspended with cytoplasmic lysis buffer containing 0.5 mM DTT and a protease and phosphatase inhibitor cocktail, incubated on ice and mixed using a 26 G needle. The lysates were centrifuged at 8000 × *g* for 20 min at 4  °C to collect the cytosolic fraction (supernatant) and nuclear fraction (pellet). The nuclear pellets were resuspended with nuclear extraction buffer containing 0.5 mM dithiothreitol (DTT) and a protease and phosphatase inhibitor cocktail and incubated for 1 h at 4 °C by inversion. The nuclear fraction was collected by centrifugation (16,000 × *g*, 5 min, 4 °C). The proteins of the cytosolic and nuclear fractions were analysed by co-IP following the protocol. Each experiment was done on three biological replicates with two different cell lines.

### Cell transfection and RNA knockdown (KD)

Transient transfection with small interfering RNA (siRNA) was done in the presence of HiPerFect transfection reagent (Qiagen) according to the manufacturer’s instructions. siRNA oligonucleotides (sequences provided in Supplemental Table 3) were obtained from Qiagen (Silencer Select NF-κB, SMAD2, SMAD4, TAK1; Allstars siRNA Negative Control). NB cells were transfected with 10 nM of siRNA over 48 h. The efficiency of siRNA on gene KD was verified in cell lysates by WB. Two target siRNAs (single or combined) and one scrambled sequence were used for each gene KD. Each experiment was done on three biological replicates with two different cell lines.

### Immunofluorescence

Neuroblastoma cells were plated in 8-well chamber slides (Tissue-Tek) and cultured in IMDM supplemented with 2% Ac-FBS (v/v) and rTGF-β1 or in the presence of CM from triple co-cultures, with or without the TGF-βR1 inhibitor, Galunisertib. The next day, the cells were fixed with 4% (w/v) paraformaldehyde in PBS for 5 min, rinsed in PBS, and blocked with 15% (v/v) FBS in PBS for 10 min. Cells were incubated overnight at 4 °C with primary antibodies as indicated (Supplementary Table 2). The next day, the slides were washed three times for 5 min with PBS-0.1% (v/v) Tween-20 and incubated with a fluorochrome-conjugated secondary antibody for 45 min at room temperature. Slides were washed in PBS-0.1% Tween-20 and mounted with Vectashield mounting medium containing 4,6-diamino-2-phenylindole (DAPI) (Vector Laboratories). Cells incubated without primary antibodies were used as negative controls. Fluorescence images were acquired on a DMI6000B microscope equipped with a 40×/1.25 oil HC PLAN APO Ph2 lens (Leica Microsystems) and ORCA-Flash 4.0 LT camera (Hamamatsu Corporation). The system was controlled by the Leica LAS X 3.6.0 software. Quantification of the nuclear translocation of phosphoproteins was obtained by counting nuclei p-SMAD2 or p-NF-κB positive cells in four microscopic fields from four biological replicates, using ImageJ software (NIH). Data were graphed in GraphPad Prism.

### Immunohistochemistry

Immunohistochemistry for p-NF-κB p65 was performed as previously described on patient-derived tumours (Children’s Hospital Los Angeles IRB: CCI-06-00198) and on tumours generated from the orthotopic implantation in immunodeficient NOD SCID NGS Gamma (NGS) mice of NB cells (CHLA-255-LUC or CHLA-136-LUC) with MN and/or MSC (Protocol #41–23) that were used in a previous publication (18) (Supplementary Table 2).

### Drug response xenotransplanted model

Animal experiments were performed in accordance with a protocol approved by the Institutional Animal Care Utilisation Committee at The Saban Research Institute of Children’s Hospital Los Angeles (Protocol #41–23 approved on October 25, 2023). A drug response protocol was developed (Fig. S5B). Eight-to twelve-week-old NSG mice, both males and females, were divided into three groups of three to five mice each and subcutaneously implanted with SK-N-BE(2)-LUC cells (1.2  ×  10^6^ cells) alone or mixed with MN and MSC (TME) (ratio 4:4:1 for NB:MN:MSC) in a total volume of 100 µL. Mice treated with Etoposide (5 mg/kg) received intra-peritoneal injections every two days on days 6, 8 and 10. Mice treated with Galunisertib received oral gavage with 50 mg/kg of Galunisertib suspended in 1% carboxymethylcellulose sodium salt, 0.085% povidone (R&D Systems), 0.5% SDS (Bio-Rad) and 0.05% antifoam L-30 emulsion (Sigma) during seven days (days 5–11). Following tumour implantation, mice were monitored before and after treatment using bioluminescence imaging (Xenogen IVIS 100, Caliper Life Science) to measure luciferase intensity and tumour growth. Two independent experiments were done with four to six mice per  group.

### Statistical analysis

Wilcoxon–Mann–Whitney test was used for overall condition effects using GraphPad Prism 9.0 Software (GraphPad). All data are presented as mean ± SD of at least three to five independent experiments with technical replicates as indicated in the legend to the figures. *p*-value < 0.05 was considered statistically significant. ****p < 0.0001, ***p < 0.001, **p < 0.01, *p < 0.05, ns not significant.

## Results

### TGF-β1 induces environment-mediated drug resistance (EMDR) in NB cells

**Fig. 1:**
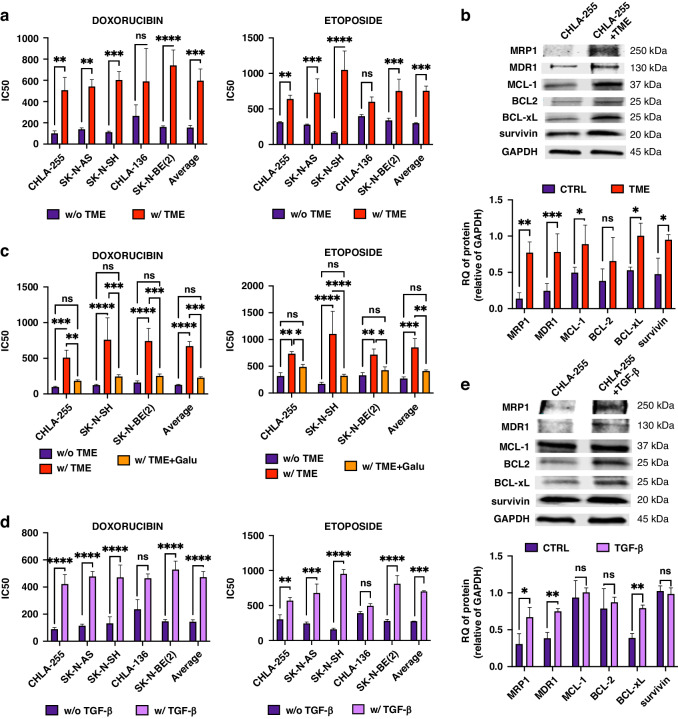
TGF-β1 induces environment-mediated drug resistance (EMDR) in NB cells. **a, c**, **d** NB cells (CHLA-255, SK-N-AS, SK-N-SH, CHLA-136, SK-N-BE(2)) were cultured alone or in the presence of MN and MSC (TME cells) (**a**); with or without Galunisertib (Galu, TGF-βR1 inhibitor, 5 μM) (**c**); or in presence of 1500 pg/ml of rTGF-β1 (**d**); for 24 h before being treated with concentrations of Doxorubicin (DOX) or Etoposide (ETOP) ranging from 10 nM to 20 μM for 72 h. After treatment, cell viability was evaluated by Trypan Blue exclusion. The graphs represent the mean ± SD of IC50 from three independent experiments with technical duplicates for each NB cell lines. **b, e** WB analysis of lysates of NB cells (CHLA-255, SK-N-BE(2)) cultured as indicated in panel a and d for the indicated proteins. *Top*: representative image (CHLA-255 lysates) of one among four blots. *Bottom*: Quantitative analysis by scanning. The data are expressed as the mean ± SD of the indicated protein:GAPDH ratio from four (**b**) or five (**e**) separate experiments. The *p*-values were determined by Wilcoxon–Mann–Whitney test. ****p < 0.0001, ***p < 0.001, **p < 0.01, *p < 0.05, ns not significant.

We first evaluated whether the TME could affect the drug sensitivity of NB cells by co-culturing human MN and human bone marrow-derived MSC (together designated TME cells) in the presence of NB cells (ratio of 4:1:4 for NB:MSC:MN). We have previously shown that human MN differentiate into TAM and human MSC into CAF when in the presence of NB cells [18]. After 72 h of co-culture with TME cells, NB cells (*n* = 5) were collected and tested for their sensitivity to Doxorubicin (DOX) and Etoposide (ETOP) and compared with NB cells cultured alone. With the exception of CHLA-136, a multidrug-resistant cell line [22], there was a statistically significant increase in the inhibitory concentration (IC50) of both drugs when NB cells were cultured 72 h in the presence of TME cells vs. alone (average IC50 of 603 ± 45.9 nM vs.139.9 ± 41.9 nM for DOX, and an average IC50 for five cell lines of 731.1 ± 68.4 nM vs. 292.8 ± 67.3 nM for ETOP) (Figs. 1a and S1A). A WB analysis of NB cell lysates obtained 72 h after co-cultures revealed an increase in the expression of multidrug resistance proteins MRP1 and MDR1 and survival proteins such as BCL-xL, MCL-1 and survivin in the presence of TME cells (Fig. 1b). Because we have previously reported that the medium of co-cultures of TAM and CAF with NB cells contains high levels of total and active TGF-β1 (1785.1 ± 29.9 and 896.2 ± 61.3 SEM pg/ml, respectively) [18], to test whether TGF-β1 could be responsible for the drug resistance observed in the presence of TME cells, we performed experiments (with three cell lines) in the presence of Galunisertib, a TGF-βR1/ALK5 inhibitor, added to the co-cultures of NB and TME cells. These experiments demonstrated a statistically significant return of the IC50 to values observed in the absence of TME cells (average IC50 of 228.6 ± 21.4 nM for DOX and 407.8 ± 45.4 nM for ETOP) suggesting a contribution of TGF-β1 (Figs. 1c and S1B). The contribution of TGF-β1 to EMDR was confirmed by drug sensitivity experiments in the presence of human recombinant (r)TGF-β1 (1500 pg/ml, a concentration observed in the triple co-cultures) that indicated an increase of IC50 (average IC50 of 465.9 ± 20.2 nM for DOX and 628.9 ± 61.4 nM for ETOP) in all cell lines at the exception of CHLA-136, which was also associated with an increase in expression of MDR1, MRP1 and BCL-xL but not BCL2, MCL-1 and survivin (Fig.1d, e and S1C–S1E). Thus, the data point to TGF-β1 as the main cytokine present in the culture medium of triple NB, MN and MSC co-cultures driving EMDR.

### TGF-β1 activates NF-κB in NB cells

**Fig. 2:**
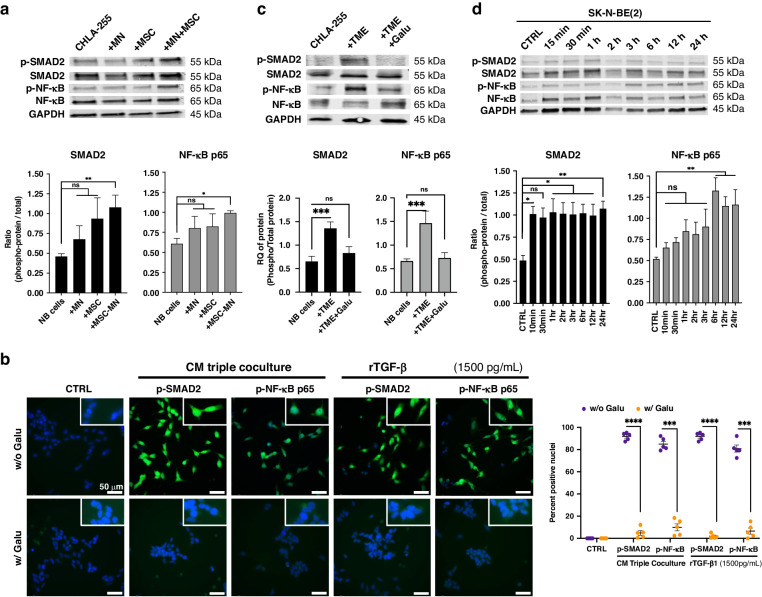
TGF-β1 activates canonical and non-canonical signalling in NB cells. **a**, **c** WB analysis of NB lysates of NB cells (CHLA-255, CHLA-136, SK-N-BE(2)) cultured alone or in presence of MN, MSC or their combination (TME cells) for 72 h (**a**), and in presence or in absence of Galunisertib (Galu, 5 μM) (**c**). Top: representative image (CHLA-255 lysates) of one among three blots. Bottom: Quantitative analysis by scanning. The data represent the mean ± SD of the indicated p-protein:protein ratio from three separate experiments. **b** NB cells (CHLA-255, CHLA-136) were cultured alone in regular medium, in CM (75% CM/25% regular medium) from triple co-cultures, or in presence of rTGF-β1 and examined by immunofluorescence for p-SMAD2 and p-NF-κB as indicated in Material and methods. Galunisertib was added for 24 h prior to analysis when indicated. Left: Representative photomicrographs of CHLA-255 (bar = 50μm). Right: The data represent the mean ± SD number of positive nuclei from four microscopic fields in four independent experiments. **d** Time course analysis of SMAD2 and NF-κB activation by WB of lysates from NB cells (CHLA-255, SK-N-BE(2)) treated with rTGF-β1 (1500 pg/ml). Top: representative image (SK-N-BE(2) lysates) of one among two blots. Bottom: Quantitative analysis by scanning. The data represents the mean ± SD of the indicated p-protein:protein ratio from three separate experiments. The *p*-values were determined by Wilcoxon–Mann–Whitney test. ****p < 0.0001, ***p < 0.001, **p <0.01, *p < 0.05, ns not significant.

The contribution of TGF-β1 to multidrug resistance is presently unclear. An absence of correlation between TGF-β1 levels and multidrug resistance was reported in chronic lymphocytic leukaemia [23] and an enhancement of MDR1 gene expression was described in a human leukaemia cell line [24]. In contrast, MRP1 and MDR1 and BCL-xL have been previously reported to be upregulated by NF-κB [25–27]. We therefore looked for evidence of NF-κB activation in NB cells cultured in the presence of MSC and MN. Interestingly, in addition to the anticipated increase in phosphorylated (p)-SMAD2, we observed an increase in p-NF-κB p65 by WB (Fig. 2a) and an increase in p-SMAD2 and p-NF-κB nuclear translocation by immunofluorescence microscopy (Fig. 2b) in NB cells in triple co-cultures or in NB cells cultured in the presence of CM from triple co-cultures. These effects were suppressed by the addition of Galunisertib, pointing to a central role of TGF-β1 (Fig. 2c). Consistently, we demonstrated that the treatment of NB cells with rTGF-β1 (1500 pg/ml for 72 h) increased the phosphorylation of NF-κB p65 and SMAD2 over time for at least 24 h (Fig. 2d).

### NF-κB activation by TGF-β1 is dependent on TAK1

**Fig. 3:**
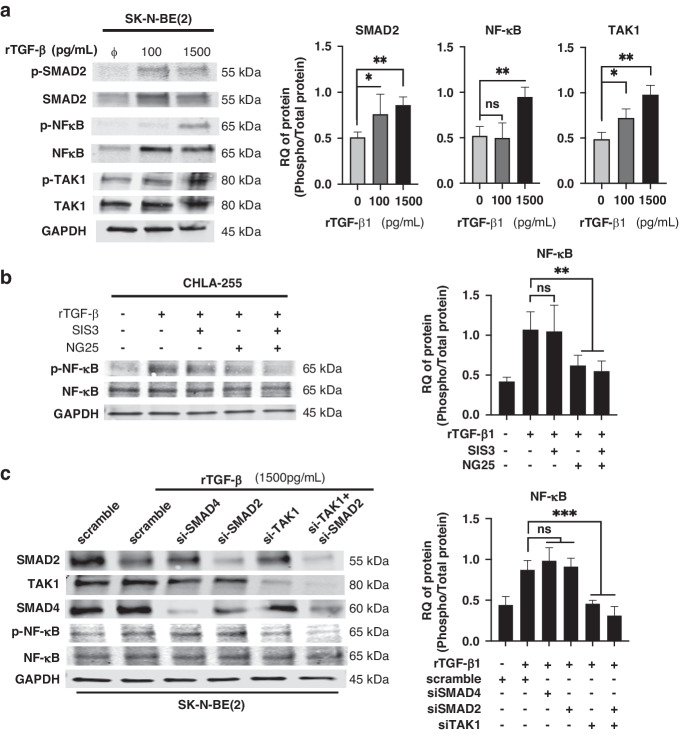
NF-κB activation by TGF-β is dependent on TAK1, but not SMAD2. **a** WB analysis of indicated proteins from lysates of NB cells (CHLA-255, CHLA-136, SK-N-BE(2)) cultured in presence or absence of rTGF-β1 for 72 h. *Left*: representative image (SK-N-BE(2) lysates) of one among five blots. *Right*: Quantitative analysis by scanning. The data are expressed as the mean ± SD of the indicated p-protein:protein ratio from five separate experiments. **b** WB analysis of NB cells (CHLA-255, SK-N-BE(2)) treated for 72 h as indicated and examined by WB. Left: representative image (CHLA-255 lysates) of one among four blots. *Right*: The data represents the mean ± SD of the indicated p-protein:protein ratio from four separate experiments. **c** NB cells (CHLA-255, SK-N-SH, SK-N-BE(2)) were transiently transfected with the indicated siRNA and treated with rTGF-β1 for 72 h and examined by WB for the indicated proteins. Left: representative image (SK-N-BE(2) lysates) of one among three blots. Right: Quantitative analysis by scanning. The data are expressed as the mean ± SD of the indicated p-protein:protein ratio from three separate experiments. The *p*-values were determined by Wilcoxon–Mann–Whitney test. ***p < 0.001, **p < 0.01, *p < 0.05, ns not significant.

TGFβ-1 signalling occurs through a canonical SMAD2/3/4 transcriptional activation pathway and a TGF-β1-activated kinase (TAK)1-mediated non-canonical pathway [28–30]. WB analysis of NB cells treated with rTGF-β1 (1,500 pg/ml) revealed an increase in p-SMAD2, p-NF-κB p65 and p-TAK1 (Fig. 3a) suggesting an involvement of both pathways. The contribution of SMAD2/3 to NF-κB activation has been previously reported but its mechanism remains unclear [31–34]. We thus performed a series of pharmacological (using small molecule inhibitors at non-toxic concentrations, Fig. S2) and genetic (siRNA) loss of function experiments in rTGF-β1-treated NB cells examining the effect of these two pathways on NF-κB activation. Inhibition of SMAD2/3 with the small inhibitor SIS3 did not affect the phosphorylation of NF-κB p65 whereas inhibition of TAK1 with the small molecule inhibitor NG25 (in the presence or absence of SIS3) blocked NF-κB p65 phosphorylation **(**Fig. 3b**)**. Consistently, KD of SMAD2 or SMAD4 by siRNA had no effect on the phosphorylation of NF-κB p65 whereas TAK1 KD (with and without SMAD2 KD) suppressed the phosphorylation of NF-κB p65 in rTGF-β1-treated NB cells (Fig. 3c). From these data we concluded that NF-κB activation by TGF-β1 was dependent of the TAK1 non-canonical pathway.

### Induction of EMDR by TGF-β1 is NF-κB and SMAD2 dependent

**Fig. 4:**
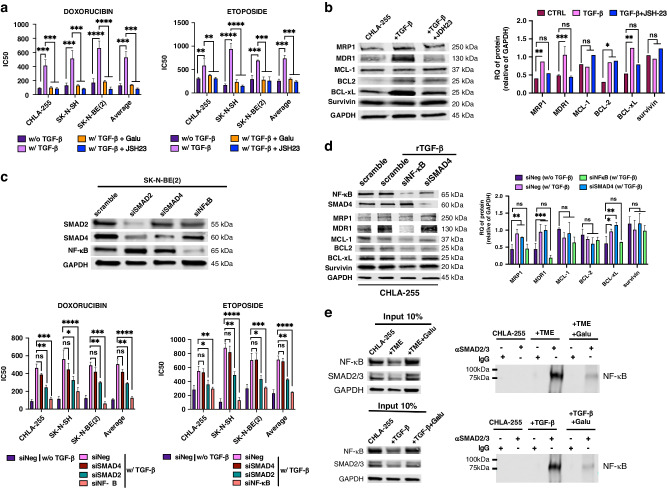
Induction of EMDR by TGF-β1 is NF-κB dependent. **a** NB cells (CHLA-255, SK-N-SH, SK-N-BE(2)) were cultured alone or with rTGFβ-1 (1500 pg/ml), in the presence or in absence of JSH23 (1 μM) or Galunisertib (Galu, 5 μM) for 24 h before being treated with Doxorubicin (DOX) or Etoposide (ETOP) during 72 h as in Fig. 1. The graphs represent the mean ± SD of IC50 from three independent experiments with a duplicate for each NB cell line. **c** NB cells (CHLA-255, SK-N-SH, SK-N-BE(2)) were transiently transfected with the indicated siRNA, treated with rTGF-β1 (1500 pg/ml) for 24 h before being treated with DOX or ETOP during 72 h and examined for viability by Trypan Blue. Top: Representative image (SK-N-BE(2) lysates) of WB analysis of the indicated proteins after transfection. *Bottom*: The graphs represent the mean ± SD of IC50 from three independent experiments with duplicates for each NB cell line. **b**, **d** NB cells (CHLA-255, SK-N-BE(2)) were treated as indicated in panel A/C and analysed by WB. Left: representative image (CHLA-255 lysates) of one among four blots. *Right*: Quantitative analysis by scanning. The data are expressed as the mean ± SD of the indicated protein:GAPDH ratio from four separate blots. **e** NB cells (CHLA-255, SK-N-BE(2)) were co-cultured with MN and MSC (TME) Top: or treated with rTGF-β1(1500 pg/ml) for 72 h Bottom: with and without Galunisertib. Lysates were immune-precipitated with an anti-SMAD2/3 antibody and examined by WB with anti-NF-κB p65 as indicated in materials and methods. Representative image (CHLA-255 lysates) of co-immunoprecipitation analysis (right) and input (10% v/v) (left) of one among three experiments. The *p*-values were determined by Wilcoxon–Mann–Whitney test. ****p < 0.0001, ***p < 0.001, **p < 0.01, *p < 0.05, ns not significant.

We next asked whether NF-κB activation by TGF-β1 was necessary for the induction of drug resistance. We first demonstrated that the increase in IC50 for DOX and ETOP observed in the presence of rTGF-β1 was reversed in the presence of Galinusertib (average IC50 of 138.4 ± 24.9 nM for DOX and 292.5 ± 42.2 nM for ETOP) and JSH23, an inhibitor of NF-κB nuclear translocation (average 84.6 ± 18.2 nM for DOX and 237.7 ± 45.9 nM for ETOP) (Figs. 4a and S3A). Blocking NF-κB p65 nuclear translocation with JSH23 inhibited the effect of rTGF-β1 on the expression of MRP1, MDR1 and BCL-xL but not BCL2, MCL-1 and survivin (Fig. 4B). In contrast, SMAD4 KD had no effect on DOX and ETOP sensitivity in the presence of rTGF-β1 (average IC50 of 417.6 ± 16.9 nM for DOX and 688.3 ± 84.4 nM for ETOP), whereas NF-κB p65 KD entirely restored the sensitivity of rTGF-β1-treated NB cells (average of 125.6 ± 40.1 nM for DOX and 268.7 ± 69.3 nM for ETOP). Surprisingly, SMAD2 KD partially but statistically significantly restored the sensitivity of rTGF-β1-treated NB cells to DOX and ETOP (average IC50 of 275 ± 46.9 nM for DOX and 428.4 ± 39.2 nM for ETOP) (Figs. 4c andS3B). Whereas SMAD4 KD had no effect on the expression of multidrug resistant and survival proteins, NF-κB p65 KD decreased the expression of MRP1, MDR1, and BCL-xL in rTGF-β1-treated cells (Fig. 4d). Having shown that SMAD2 does not affect NF-κB phosphorylation, we postulated that SMAD2 could interact with NF-κB p65 and contribute to its transcriptional activity independently of its transcriptional activity as a SMAD2/3/4 complex. Immunoprecipitation experiments demonstrated the presence of a SMAD2/3/NF-κB complex in lysates of NB cells cultured in the presence of TME cells or treated with rTGF-β1 that was suppressed in the presence of Galunisertib (Fig.  4e). This complex was detected in the cytoplasm and the nucleus suggesting that its formation preceded its nuclear translocation (Fig. S3C). Thus, the data indicates that TGF-β alters the sensitivity of NB cells to DOX and ETOP and the expression of MRP1, MDR1 and BCL-xL through a TAK1/NF-κB/SMAD2/3 pathway with TAK1 phosphorylating NF-κB and SMAD2 contributing to the NF-κB transcriptional complex.

### NF-κB activation by TGF-β drives the production of IL-6 family of cytokines that contribute to EMDR

**Fig. 5:**
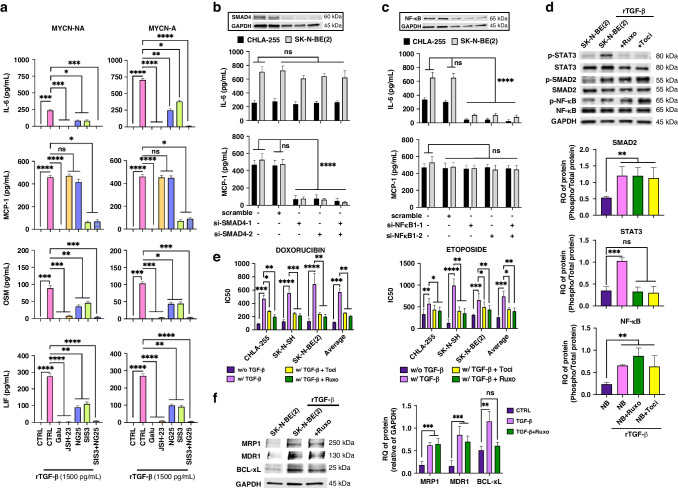
NF-κB activation by TGF-β drives the production of IL-6 family of cytokines that contribute to EMDR. **a** MYCN-NA NB cells (CHLA-255, SK-N-AS) (*left*) and MYCN-A NB cells (CHLA-136, SK-N-BE(2)) (*right*) were cultured alone or with rTGF-β1 (1500 pg/ml), in presence or in absence of Galunisertib (5 μM), JSH23 (1 μM), NG25 (1 μM) or SIS3 (2 μM). After 72 h the culture medium was examined for the presence of indicated cytokines. The data represents the mean ± SD concentrations of indicated cytokines from three (CHLA-255, SK-N-BE(2)) and two (SK-N-AS and CHLA-136) independent experiments done in technical duplicates. **b**, **c** NB cells (CHLA-255, SK-N-BE(2)) were transiently transfected with SMAD4 and NF-κB siRNA and treated for 24 h with rTGF-β1 (1500 pg/ml) before the medium was examined for IL-6 and MCP-1. *Top*: Representative image of WB analysis of the indicated proteins after transfection. Bottom: The graph represents the mean ± SD IL-6 and MCP-1 concentrations from three independent experiments done in technical duplicates. **d**, **f** WB analysis of lysates of NB cells (CHLA-255, SK-N-BE(2)) cultured with rTGF-β1 (1500 pg/ml) in presence or in absence of Tocilizumab (Toci, 10 μg/ml) or Ruxolitinib (Ruxo, 1 μM) for the indicated proteins. Top: representative image (SK-N-BE(2) lysates) of one among three blots. Bottom: Quantitative analysis by scanning. The data are expressed as the mean ± SD of the indicated p-protein:protein or protein:GAPDH ratio from three separate experiments. **e** NB cells (CHLA-255, SK-N-SH, SK-N-BE(2)) were cultured alone or with rTGF-β1(1500 pg/ml), in presence or in absence of Tocilizumab or Ruxolitinib, for 24 h before being treated with Doxorubicin (DOX) or Etoposide (ETOP) for 72 h before being evaluated for viability by Trypan Blue. The graphs represent the mean ± SD of IC50 from three independent experiments with duplicate for each NB cell line. The *p*-values were determined by Wilcoxon–Mann–Whitney test. ****p < 0.0001, ***p < 0.001, **p < 0.01, *p < 0.05, ns not significant.

TGF-β1 induces in NB cells the expression of several cytokines such as IL-6 and MCP-1 [18]. We show that TGF-β1 induces the production of IL-6, MCP-1 but also OSM and LIF by NB cells. MCP-1, OSM and LIF production did not differ between MYCN-A and MYCN-NA NB cells. On the contrary, there was a difference in IL-6 production between MYCN-A and MYCN-NA NB cells (average of 705.1 ± 22.1 and 236.9 ± 15.1 SD pg/mL, respectively). We thus asked whether the production of these cytokines by TGF-β1 was – like MRP1, MDR1 and BCL-xL – dependent on NF-κB p65 activation. Blocking NF-κB nuclear translocation with JSH23 in rTGF-β1-treated cells suppressed the production of IL-6, and LIF and OSM, members of the IL-6 family, but not MCP-1. Blocking SMAD2/3 with SIS3 or TAK1 with NG25 partially inhibited the expression of IL-6, LIF and OSM, and their combination completely suppressed the production of these cytokines. In contrast the production of MCP-1 by TGF-β1 was not inhibited by blocking TAK1 but by blocking SMAD2/3 (Figs. 5a andS4A). Consistently, NF-κB p65, TAK1 and SMAD2 KD but not SMAD4 KD affected partially or totally the production of IL-6, LIF and OSM whereas MCP-1 expression was not affected by NF-κB p65 and TAK1 KD but by SMAD4 and SMAD2 KD (Figs. 5b, c and S4B–S4F).

The data thus indicated a dependency of TGF-β1-induced IL-6, LIF and OSM on TAK1 and SMAD2-mediated NF-κB p65 activation as observed for MDR1, MRP1 and BCL-xL, whereas in the case of MCP-1, induction by TGF-β1 was independent of NF-κB p65 activity. IL-6 has been reported by us [16] and others [35] to promote drug resistance by activating STAT3 and the expression of survival factors like BCL-xL, MCL-1 and survivin. We thus postulated that the upregulation of IL-6 by TGF-β1 could create an autocrine loop by which IL-6 activating STAT3 in TGF-β1-treated cells could contribute to EMDR by the upregulation of survival factors like BCL-xL. We first demonstrated that blocking STAT3 activation by inhibiting IL-6 binding to its receptor with Tocilizumab or blocking JAK2 activation with Ruxolitinib, had no effect on SMAD2 and NF-κB p65 phosphorylation in TGF-β1-treated NB cells (Fig. 5d) but partially restored sensitivity to DOX and ETOP (Figs. 5e andS4G) and inhibited the expression of BCL-xL but not MDR1 and MRP1 (Fig. 5f). Thus, the data point to STAT3 as a downstream target of TGF-β1 through the production of IL-6 by the TGF-β1/TAK1/NF-κB/SMAD2 pathway, therefore contributing to TGF-β1-induced EMDR.

### Evidence for NF-κB activation in xenotransplanted NB tumours infiltrated with TAM and CAF and in patient-derived tumours

**Fig. 6:**
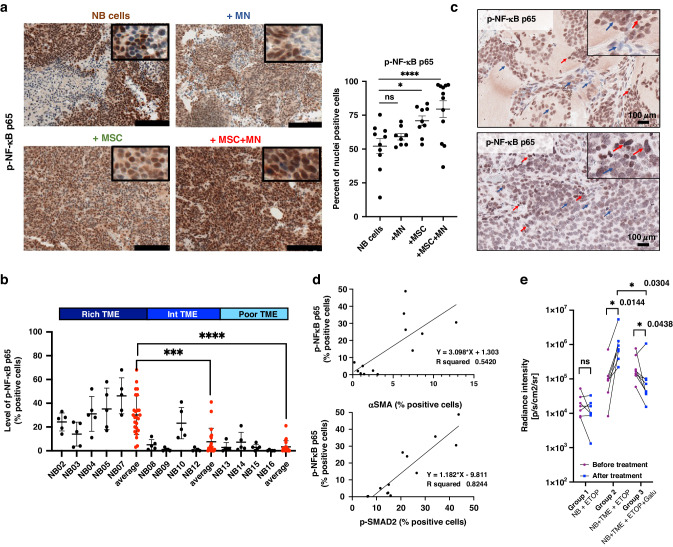
NF-κB is activated in NB tumours infiltrated with TAM and CAF. **a** Xenotransplanted tumours from subcutaneous injections of human NB cells (CHLA-255-LUC) alone or in combination with human MN and MSC were examined by immunohistochemistry (IHC) as described in Materials and Methods. *Left*: Representative photomicrographs of tumour sections for p-NF-κB p65 (bar = 100 μm). *Right*: the data represent the mean ± SD percent of nuclei positive for p-NF-κB p65 in NB cells counted in four microscopic fields from three sections obtained from two tumours in each group. **b** The graph represents the percent of positive cells for p-NF-κB p65 identified by multiplex IHC on 13 human tumour samples as indicated in Materials and Methods. The data represent the mean ± SD per cent of positive cells in five microscopy fields per tumour. **c** Representative digital microphotographs of NB tumour sections stained for p-NF-κB p65 (bar = 100 μm). Red arrows indicate a positive nuclear staining for p-NF-κB p65, and blue arrows indicate a negative or low nuclear staining. **d** The graphs represent the correlation between the percent of αSMA+ CAF or p-SMAD2+ cells, and the percent of p-NF-κB+ cells, from 13 human tumours shown in panel B. **e** NSG mice were subcutaneously implanted with SK-N-BE(2)-LUC NB cells alone or in presence of human MN and MSC (TME). Tumour growth was monitored by bioluminescence. The data represent the radiance intensity of each mouse from two independent experiments, monitored before and after treatment with ETOP with and without Galu. The statistics are calculated on the average of all mice. The *p*-values were determined by Wilcoxon–Mann–Whitney test. ****p < 0.0001, ***p < 0.001, *p < 0.05, ns: not significant.

We had previously observed an increased expression of p-SMAD2 in tumours generated in NSG mice by the co-injections of human NB cells and human MN and MSC [18]. Tissue sections of these tumours were obtained and analysed for the presence of p-NF-κB p65 by immunohistochemistry (IHC). This analysis revealed an increased expression of p-NF-κB p65 in tumours co-injected with NB cells and with MSC or with MSC and MN (Figs. 6a and S5A). We similarly examined sections of 13 primary untreated human NB tumours we had previously examined for the presence TAM and CAF [18], for nuclear p-NF-κB p65 by IHC. The data revealed the presence of nuclear p-NF-κB p65 in NB cells in TME-rich (>10% TME cells) tumours (average 30.12 ± 3.3%) compared with TME-poor (< 5% TME cells) tumours (average 3.25 ± 1.2%) or TME intermediate (TME cells between 9 and 6%) tumours (average 7.5 ± 2.6%) (Fig. 6b, c). Consistent with a contribution of TME cells and TGF-β1 in NF-κB p65 activation, we observed a statistically significant positive correlation between the level of NF-κB phosphorylation and the presence of αSMA+ CAF (*r*^2^ = 0.54), and p-SMAD2 (*r*^2^ = 0.82) (Fig. 6d). To validate the effect of TGF-β on drug resistance demonstrated in vitro, we established a new xenograft model of drug response combining Galunisertib and ETOP in immunodeficient mice (Fig. S5B). The data (Figs. 6e and S5C) demonstrate a lack of response to ETOP in NB tumours implanted in the presence of human TME cells (MN and MSC) that was reversed by adding Galinusertib during the treatment with ETOP. The data thus provide an important in vivo validation of our concept.

## Discussion

By identifying a TGF-β1/TAK1/NF-κB/SMAD2 signalling pathway triggered in NB cells by their interaction with TAM and MSC/CAF, our data brings a new insight into the contribution of TGF-β1 to tumour progression and therapeutic resistance in this cancer. The role of TGF-β1 in cancer is complex and includes anti-tumour activities mainly reported in the early stages of cancer development as well as pro-tumour and immunosuppressive activities seen in later stages of cancer progression [36, 37]. TGF-β1 is a known inducer of epithelial to mesenchymal transformation (EMT) and its effect in cancer has been associated with chemo- [38] and radio-therapy resistance [39] although the mechanisms are not entirely known [40]. In NB, TGF-β1 is spontaneously expressed at low levels (in the 100 pg/ml range) by NB cells, and its effect on proliferation and differentiation has been the subject of several previous publications reporting different effects in various NB cell lines [41]. It is induced by retinoic acid (RA) creating a negative autocrine cell growth regulatory loop needed for differentiation [42] and its down-regulation by MYC-N contributes to RA resistance [43]. It is upregulated in the TME by TAM and CAF and it suppresses the cytotoxic activity of natural killer (NK) cells [18, 44, 45]. Its effect on NB cells is a function of the receptors expressed. In general, in NB cells expressing TGF-βR1 and R2, exposure to TGF-β1 stimulates the cell cycle and increases the expression of anti-apoptotic proteins, whereas in TGF-βR3 expressing NB cells TGF-β1 inhibits proliferation and promotes differentiation [46–48]. The role of TGF-β1 in drug resistance in NB has not been explored and to our knowledge, this is the first report identifying a mechanism by which TGF-β1 promotes EMDR in NB.

Our data demonstrate that drug resistance induced by TGF-β1 requires activation of NF-κB. Activation of NF-κB by TGF-β1 has been described in models of bleomycin-induced pulmonary fibrosis [49] and in osteoclasts, where it activates TAK1/MEK/AKT/NF-κB to promote their differentiation and survival [50]. Here we demonstrate that NF-κB activation by TGF-β1 is dependent on the non-canonical TAK1-dependent pathway [29]. Surprisingly, experiments with a small inhibitor of SMAD2/3 and genetic KD experiments with SMAD2 and SMAD4 indicated a contribution of SMAD2 but not SMAD4 to NF-κB-mediated drug resistance. Consistent with SMAD2 being a transcription factor, we show no effect of SMAD2 KD on phosphorylation of NF-κB p65 but demonstrate the presence of a SMAD2/NF-κB complex in the cytoplasm and nucleus in TGF-β1-treated NB cells suggesting it could enhance the transcriptional activity of the NF-κB complex. The exact mechanism of direct interaction remains unknown and will require further investigation but the detection of SMAD2/3/NF-κB complexes in the cytoplasm and the nucleus indicates that the formation of the complex does not require DNA binding.

The TGF-β1/TAK1/NF-κB/SMAD2 pathway reported here induces chemotherapy resistance via two well-known mechanisms of resistance reported in NB, upregulation of ABC transporter proteins MDR1 and MRP1 and of survival proteins like BCL-xL, MCL-1, BCL2 and survivin. Whereas all upregulated in NB co-cultured with TME cells, MCL-1, BCL2 and survivin were not upregulated in TGF-β1-treated NB cells and their expression were not affected upon pharmacological or genetic inhibition of the TGF-β1/TAK1/NF-κB/SMAD2 signalling pathway, suggesting a different mechanism of regulation. We also show that whereas the upregulation of MRP1 and MDR1 is a direct target of NF-κB activity as reported by others in breast cancer [51], the upregulation of BCL-xL is an indirect target via the autocrine IL-6/STAT3 loop as reported in liver cirrhosis [52]. We have previously reported that IL-6 produced by MSC and CAF in the TME induces the expression of BCL2, MCL-1 and survivin causing an increase in NB growth and resistance to drugs like Topotecan and Melphalan [16, 35, 53, 54]. IL-6 being downstream of the TGF-β1/TAK1/NF-κB/SMAD2 signalling pathway, one would expect an increase in expression upon treatment with rTGF-β1, which was not the case. Although the reason for this is not entirely clear, a likely explanation is an IL-6 dose-dependency of the expression of these proteins. The concentrations of IL-6 observed in rTGF-β1-treated NB cells ranged between 250 pg/ml (MYCN-NA cells) and 700 pg/ml (MYCN-A cells) cells whereas concentrations in the range of 6000 to 8000 pg/ml are observed in co-cultures of NB cells and TAM and CAF [18]. This point illustrates the complex cooperation that exists between cytokines in the TME and the role of TGF-β1 independent exogenous sources of IL-6 in promoting EMDR.

Drug resistance in NB has been recently shown to be associated with epigenetically driven adrenergic to mesenchymal transition (AMT) characterised by the loss of expression of neuroendocrine genes and gain of expression of mesenchymal genes [55–57]. The known function of TGF-β1 on EMT raises the question whether changes observed on drug-resistant proteins are associated with an increased expression of a mesenchymal phenotype, a question presently investigated by our laboratory.

The correlation between NF-κB p65 activation and αSMA+ cells and p-SMAD2 in primary NB tumours and the more abundant presence of p-NF-κB in TME-rich tumours provides support to our in vitro data. The presence of TAM and CAF with evidence of activation of the TGF-β1/TAK1/NF-κB/SMAD2 pathway in NB tumours could thus be a biomarker identifying tumours at higher risk of developing drug resistance. This possibility should be verified by analysing refractory or resistant tumours. Our in vivo data support the claim that targeting the TGF-β1/TAK1/NF-κB/SMAD2 pathway could be a valuable approach to circumvent drug resistance. Drugs and biological agents targeting the TME have increasingly taken part in the therapeutic arsenal against cancer often in combination with chemotherapy. Numerous small molecule inhibitors of the signalling pathway identified in this paper have been developed and tested in preclinical mouse models and have shown efficacy [44, 58–61]. Some like Bortezomib, a proteasome inhibitor, have been tested in clinical trials in NB showing safety and efficacy [62]. The small molecule TGFβ inhibitor Galunisertib has been tested in several clinical trials in patients with cancer, either alone or in combination with Lomustine or anti-PDL-1 with close to 50% experiencing treatment-related adverse effects [63, 64]. It was discontinued in 2020. In our in vivo drug response model, we have used Galunisertib for a short period (7 days) during the administration of chemotherapy, a schedule that may not be associated with the toxicity observed in more prolonged administration as in the past clinical trials. Thus, the use of Galunisertib or similar inhibitors should be revisited within the context of EMDR as illustrated in this paper. These agents could have an important role in combination with chemotherapy or immunotherapy in NB tumours showing high infiltration with TAM and CAF cells and evidence of TGF-β1 and NF-κB activity in NB cells. In addition to their effect on drug resistance, by targeting TGF-β1 these inhibitors will contribute to inhibiting immune escape in immunotherapy.

In summary, the identification of a TGF-β1/TAK1/NF-κB/SMAD2 signalling pathway driven by the interaction between NB cells, TAM, and CAF in the TME leading toward drug resistance, provides an opportunity to revisit the contribution of TGF-β1 and NF-κB in NB progression.

### Supplementary information

supplemental table

supplemental figures

## Data Availability

The data that support the findings of this study are available from the corresponding author, [YAD], upon reasonable request.
