# Supplementary material for: Nuclear factor-κB activation by transforming growth factor-β1 drives tumour microenvironment-mediated drug resistance in neuroblastoma
Source: Br J Cancer. 2024 May 28;131(1):90–100. doi: 10.1038/s41416-024-02686-8 (PMC11231159; doi:10.1038/s41416-024-02686-8)
Supplement: Supplementary file 1 — supplemental table [file 41416_2024_2686_MOESM1_ESM.docx]

**Supplemental Table 1. List of drugs and recombinant protein**

| *Application* | Name | Target | [conc] | Reference | Company |
| --- | --- | --- | --- | --- | --- |
| Drugs | Galunisertib  (LY2157299) | TGF-βR1 (ALK5) | 5 μM | S2230 | Selleckchem |
|  | JSH-23 | NF-κB nucleus translocation | 1 μM | S7351 | Selleckchem |
|  | SIS3 | SMAD2/3 | 2 μM | S7959 | Selleckchem |
|  | NG25 | TAK1 | 1 μM | S8868 | Selleckchem |
|  | Tocilizumab  (Actemra) | IL-6R | 10 μg/mL | A2012 | Selleckchem |
|  | Ruxolitinib  (Jakafi/Jakavi) | JAK2 | 1 μM | S1378 | Selleckchem |
|  | Doxorubicin  (Adriamycin) | - | 10 to 20000 nM | NDC 0069-4030-01 | Pfizer |
|  | Etoposide  (Vepesid) | - | 10 to 20000 nM | NDC 0703-5653-01 | Teva Pharmaceuticals |
| Human recombinant protein | rTGF-β1 | TGF-β1 | 100, 600, 1500 pg/mL | 240-B-002 | R&D System |

**Supplemental Table 2. List of antibodies used**

| **Application** | **Target** | **Reference** | **Dilution** | **Company** |
| --- | --- | --- | --- | --- |
| Western Blot | GAPDH | GTX627408 | 1:2000 | GeneTex |
|  | NF-κB p65 | 8242 | 1:1000 | Cell Signaling |
|  | p-NF-κB p65 (ser536) | 3033 | 1:500 | Cell Signaling |
|  | SMAD2 | 3103 | 1:1000 | Cell Signaling |
|  | p-SMAD2 (ser255) | ab188334 | 1:500 | Abcam |
|  | SMAD4 | Sc-7966 | 1:500 | Santa Cruz |
|  | TAK1 | SAB4502922 | 1:500 | Sigma |
|  | p-TAK1 (ser412) | 06-1425 | 1:500 | Millipore |
|  | STAT3 | 4904 | 1:1000 | Cell Signaling |
|  | p-STAT3 (tyr705) | 9145 | 1:1000 | Cell Signaling |
|  | MRP1/ABCC1 | 14685 | 1:1000 | Cell Signaling |
|  | MDR1/ABCB1 | Sc-13131 | 1:300 | Santa Cruz |
|  | MCL-1 | 4572 | 1:1000 | Cell Signaling |
|  | BCL-xL | 2764 | 1:1000 | Cell Signaling |
|  | BCL2 | 4223 | 1:500 | Cell Signaling |
|  | survivin | 2808 | 1:500 | Cell Signaling |
| Immunoprecipitation | SMAD2/3 | 8685 | 1:200 | Cell Signaling |
|  | NF-κB p65 | 8242 | 1:1000 | Cell Signaling |
|  | Lamin A/C | 2032 | 1:1000 | Cell Signaling |
|  | GAPDH | GTX627408 | 1:2000 | GeneTex |
| Immunofluorescence | p-SMAD2 (ser255) | ab188334 | 1:200 | Abcam |
|  | p-NF-κB p65 (ser536) | ab86299 | 1:300 | Abcam |
| Immuno-histo-chemistry | p-NF-κB p65 (ser536) | ab86299 | 1:300 | Abcam |

**Supplemental Table 3. List of target sequences used to gene interference**

| **Target** | **Target sequence** |
| --- | --- |
| SMAD4 | AAGCAGCGTCACTACCTAA |
| SMAD4 | CCCTGTTAAACAGTAGTTGTA |
| SMAD2 | CAGGTAATGTATCATGATCCA |
| SMAD2 | AAGCCGTCTATCAGCTAACTA |
| NF-κB1 p65 (ReLa) | ATGGAGTACCCTGAGGCTATA |
| NF-κB1 p65 (ReLa) | CAGGCGAGAGGAGCACAGATA |
| TAK1 (MAP3K7) | AAACCTTATAATGACGATTCA |
| TAK1 (MAP3K7) | AACGGACAGCCAAGACGTAGA |
