# Supplementary material for: Nuclear factor-κB activation by transforming growth factor-β1 drives tumour microenvironment-mediated drug resistance in neuroblastoma
Source: Br J Cancer. 2024 May 28;131(1):90–100. doi: 10.1038/s41416-024-02686-8 (PMC11231159; doi:10.1038/s41416-024-02686-8)
Supplement: Supplementary file 2 — supplemental figures [file 41416_2024_2686_MOESM2_ESM.docx]

**Supplemental Figures:**


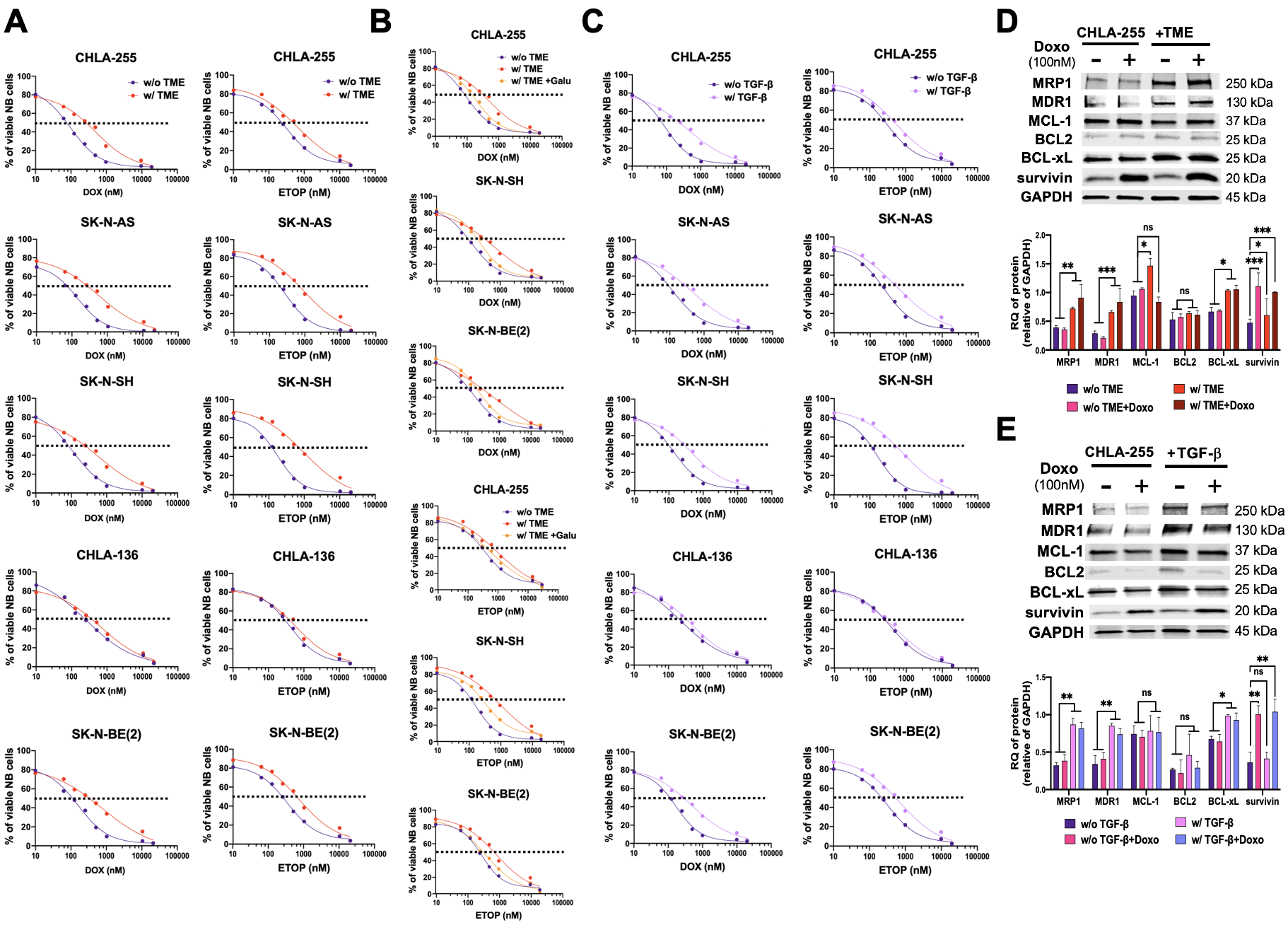


**Supplemental Figure 1**

**A, B and C.** NB cells were treated as described in Figure 1. The graphs represent the per cent viability for indicated drug concentrations. The dotted line indicates IC50. **D and E.** WB analysis of lysates of NB cells (CHLA-255) cultured as indicated in panel A and C for the indicated proteins. *Top*: representative image of one among two blots. *Bottom*: Quantitative analysis by scanning. The data are expressed as the mean ± SD of the indicated protein:GAPDH ratio from two separate experiments. The *p*-values were determined by Wilcoxon-Mann-Whitney test. ****p* <.001, **p* <.05, ns: not significant.


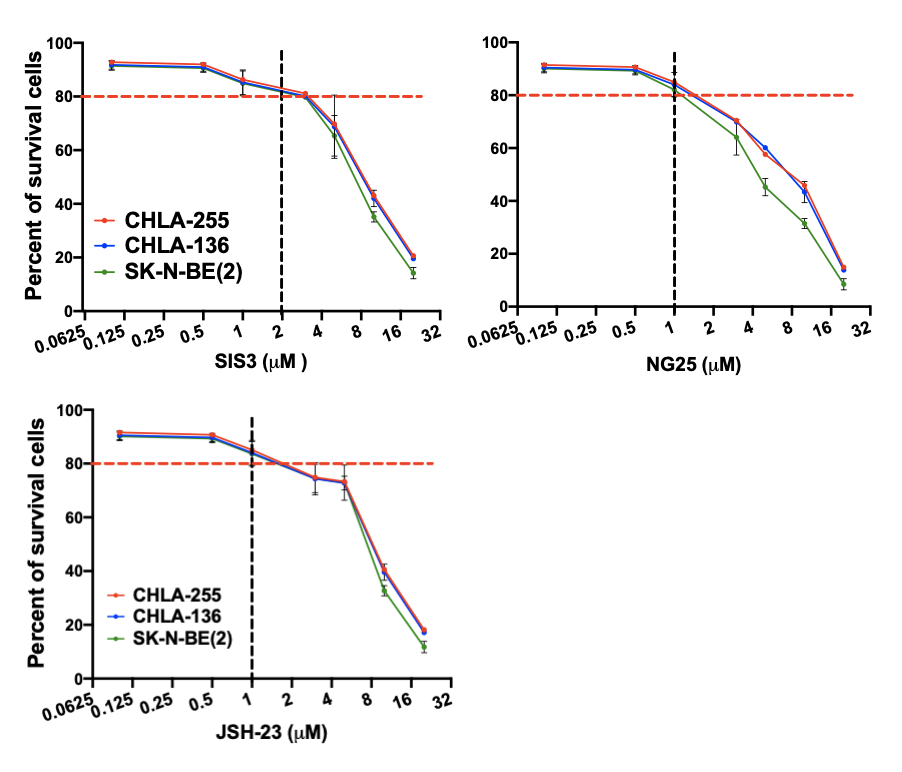


**Supplemental Figure 2**

NB cells (CHLA-255, CHLA-136, SK-N-BE(2)) were cultured in presence of increased concentrations of indicated inhibitors (10nM to 20μM) for 72h. After treatment, the viability of NB cells was evaluated by Trypan Blue. The data represent the percent of NB living cells from two independent experiment with duplicate for each NB cells. The dotted line indicates the IC80.


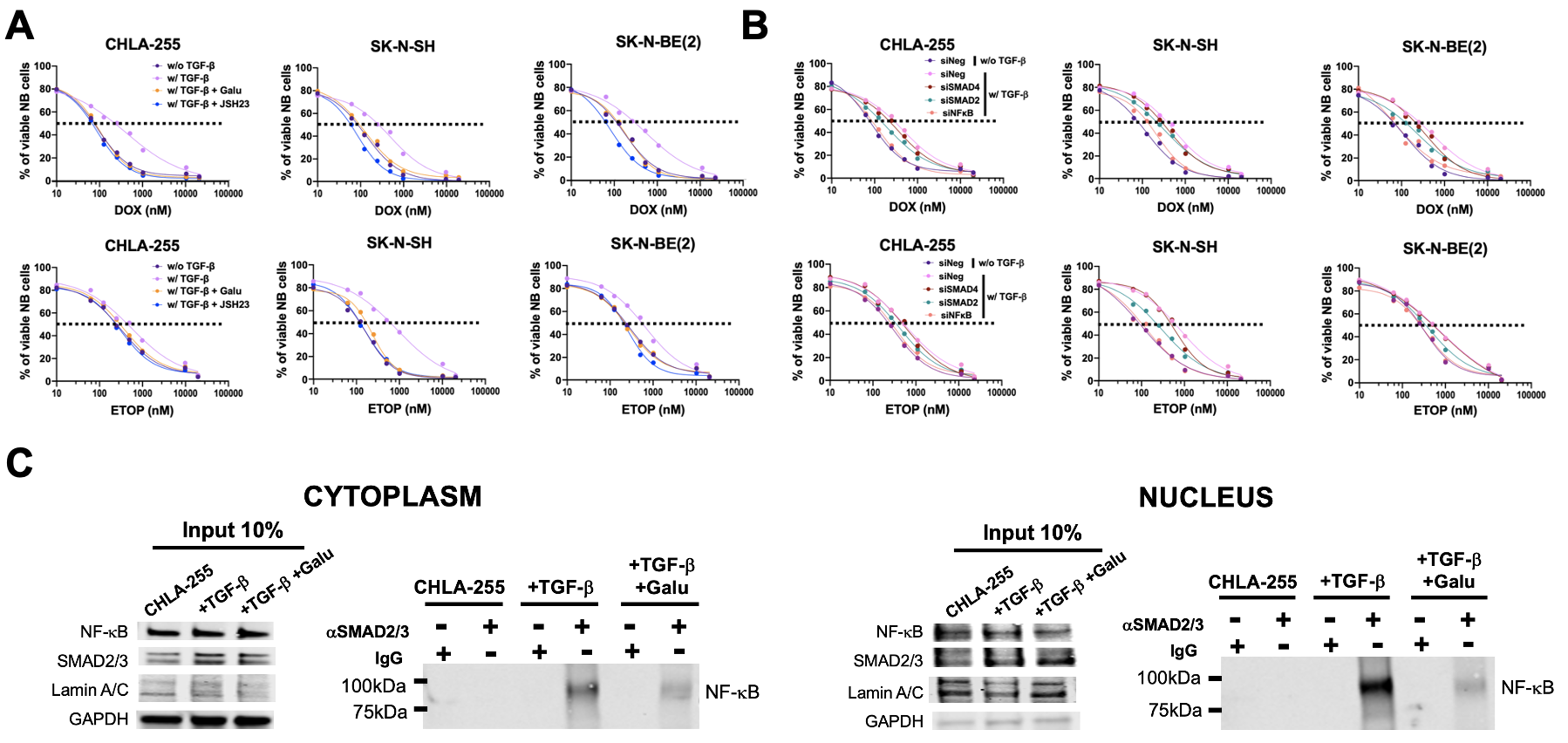


**Supplemental Figure 3**

**A and B.** NB cells were treated as indicated in Figure 4. The data represent the percent viable cells for indicated drug concentration. The dotted line represents the IC50. **C.** NB cells were treated and cytoplasm and nucleus were examined by immuno-precipitation as indicated in Figure 4 panel E. Representative image of co-immunoprecipitation analysis (CHLA-255 lysates) (right) and input (10% v/v) (left) of one among three blots from independent experiment.


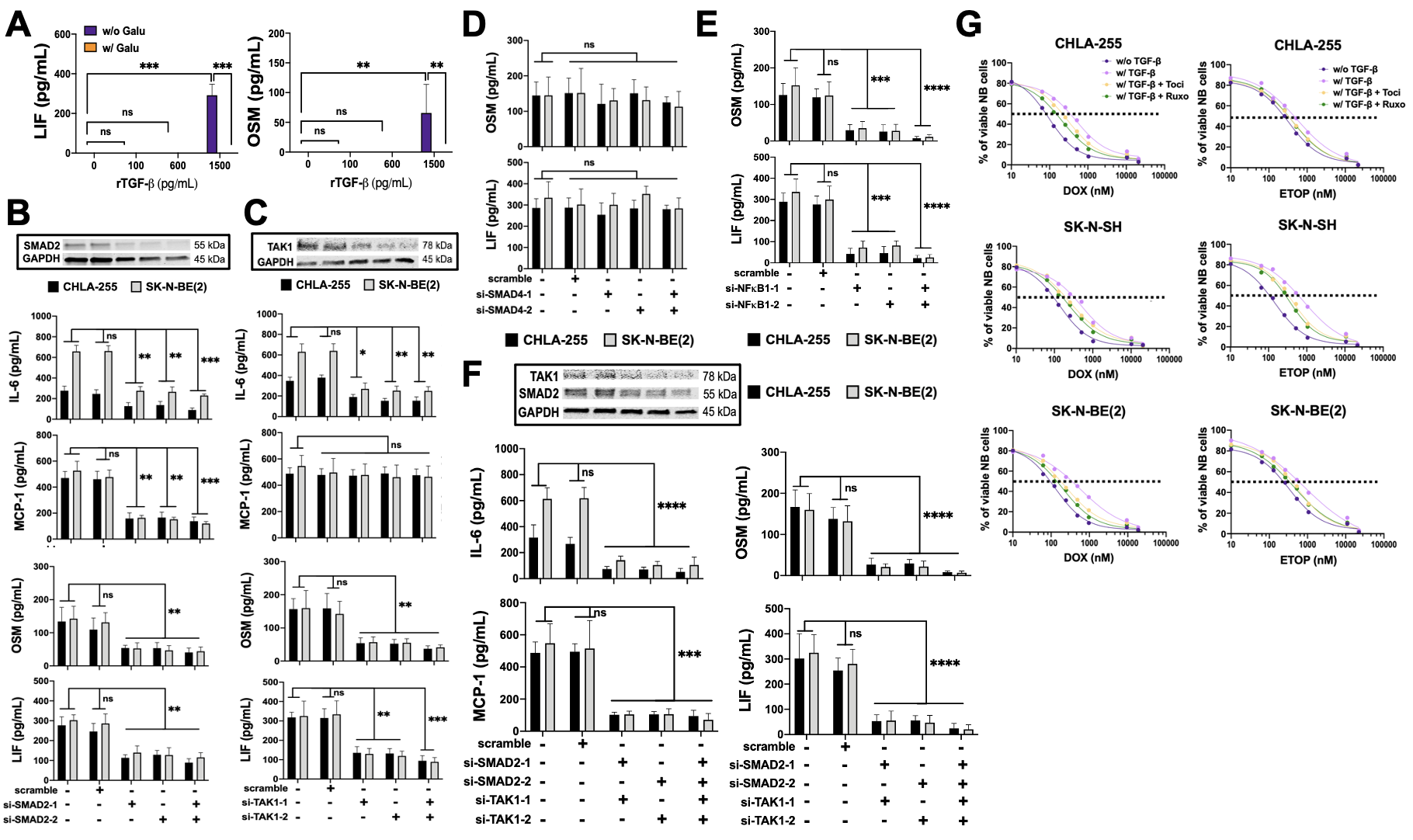


**Supplemental Figure 4**

**A.** NB cells were treated as described in Figure 5. LIF and OSM concentrations were measured in the culture medium by ELISA. **B to F.** NB cells (CHLA-255, SK-N-BE(2)) were transiently transfected with indicated siRNA and examined for the presence of indicated cytokines as described in Figure 5. *Top of each panel*: WB analysis of the indicated proteins after transfection. *Bottom of each panel*: The graph represents the mean ± SD indicated proteins concentrations from three independent experiments done in technical duplicates. **G.** NB cells (CHLA-255, SK-N-SH, SK-N-BE(2)) were treated as described in Figure 5 panel D/F. The data represent the percent of living NB cells from three independent experiment with duplicate for each NB cells. The dotted line indicates the IC50. The *p*-values were determined by Wilcoxon-Mann-Whitney test. *****p* <.0001, ****p* <.001, **p* <.05, ns: not significant.


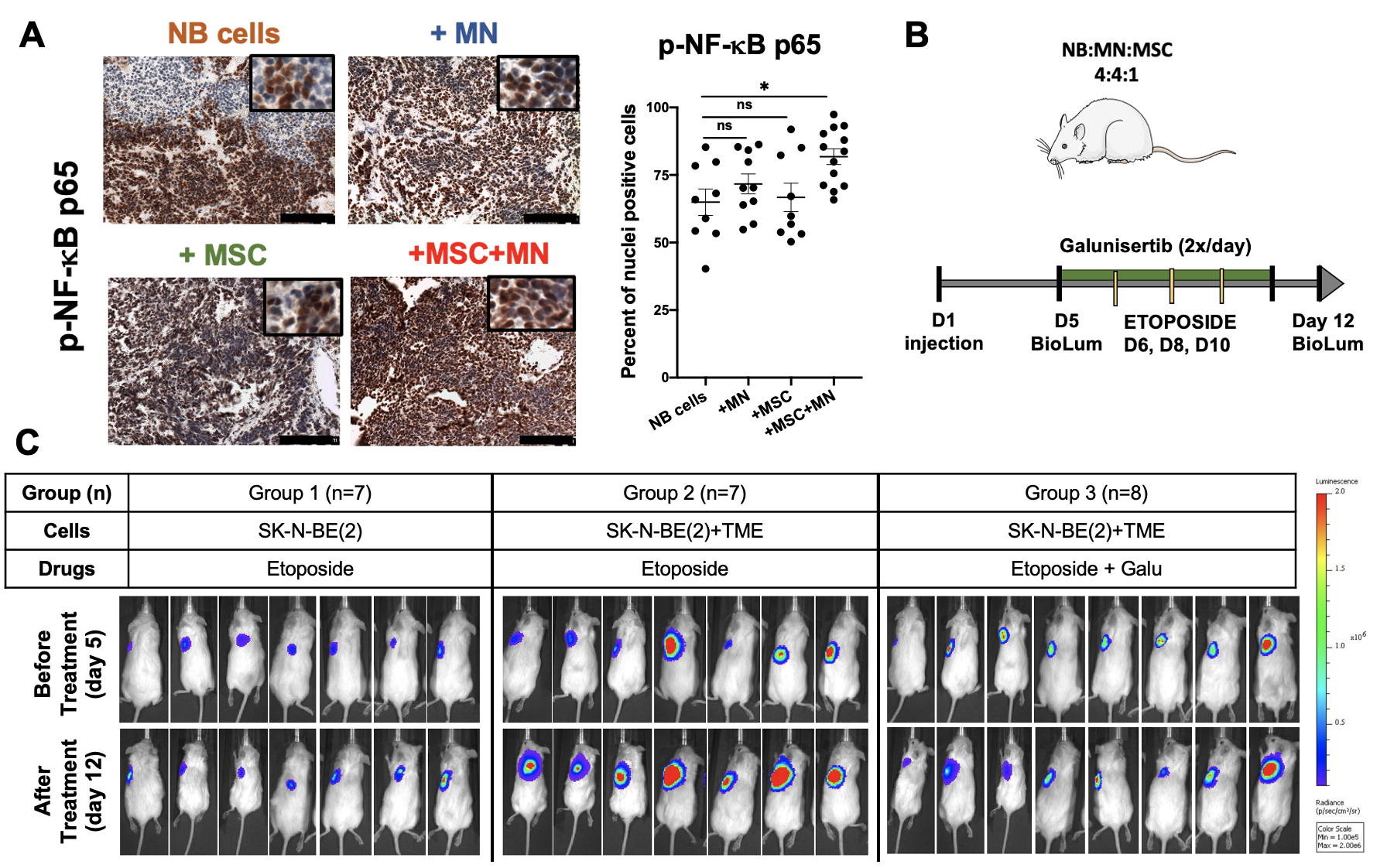


**Supplemental Figure 5**

**A.** *Left*: Representative photomicrographs of xenotransplanted NB tumors (CHLA-136-LUC) described in Figure 6 stained for p-NF-κB p65 (Bar=100um). *Right*: the data represent the mean ± SD percent of nuclei positive from NB cells counted in four microscopic fields from two or three sections obtained from two tumors in each group. The *p*-values were determined by Wilcoxon-Mann-Whitney test. **p* <.05, ns: not significant. **B.** Diagram summarizing the treatment model of immunodeficient mice that were subcutaneously implanted with NB cells (SK-N-BE(2)-LUC) in presence of TME (MN+MSC) (ratio 4:4:1, NB:MN:MSC). **C.** Bioluminescence images of each mouse bearing a SK-N-BE(2)-LUC tumor implanted and treated as indicated at the top, from two independent experiments.
